# Systematic Pan-Cancer Analysis and Experimental Verification Identify FOXA1 as an Immunological and Prognostic Biomarker in Epithelial Ovarian Cancer

**DOI:** 10.1155/2022/9328972

**Published:** 2022-11-02

**Authors:** Kai Wang, Chenan Guan, Junhui Yu, Xing Chen, Xianwen Shang, Shuangshuang Mei, Xingjun Feng, Lingzhi Zheng

**Affiliations:** ^1^Department of Obstetrics and Gynecology, Taizhou Hospital Zhejiang Province, Wenzhou Medical University, Linhai, 317000 Zhejiang, China; ^2^Department of Kidney Internal Medicine, Taizhou Hospital Zhejiang Province, Wenzhou Medical University, Linhai, 317000 Zhejiang, China; ^3^Department of Obstetrics and Gynecology, Shaoxing University, Shaoxing, 312000 Zhejiang, China

## Abstract

**Background:**

Epithelial ovarian cancer (EOC) has the lowest survival rate among female reproductive cancers present with symptoms of aggressive malignancies, poor prognosis, drug resistance and postoperative recurrence. The majority of patients with EOC are diagnosed at an advanced stage due to the therapeutic challenges including lack of early diagnosis and effective therapeutic targets for EOC.

**Methods:**

Pan-cancer analyses were performed to explore the features of forkhead-box (FOX) A1 (FOXA1) using data from TCGA and GTEx databases. R package “clusterprofiler” was used to perform the enrichment analysis of FOXA1 in EOC. Data downloaded from Drug Sensitivity in Cancer (GDSC) database were used to evaluate the association between FOXA1 and antitumor drug sensitivity. In experimental verification, FOXA1 expression was detected using qRT-PCR and western blot assays. Western blot, immunofluorescence staining, and Transwell assays were used to assess the influence of FOXA1 silencing on epithelial-mesenchymal transition (EMT) of EOC cells.

**Results:**

We found that FOXA1 was highly expressed in EOC and predicted poorer survival of EOC patients. We observed that FOXA1 expression was positively correlated EMT-related pathways. Through experimental verification, we found the underlying function of FOXA1 to promote EMT in ovarian cancers. The results from western blot, immunofluorescence staining, and Transwell assays showed that FOXA1 silencing impeded the progression of EMT and invasiveness of the cancer cells. Furthermore, CCK-8 and invasion assays suggested that siRNA-FOXA1 attenuated the ability of cancer cells to metastasize and proliferate. Dual-luciferase reporter assays confirmed the binding activity of FOXA1 to the promoter of connective tissue growth factor (CTGF). In addition, we found that FOXA1 was closely correlated immunosuppressive microenvironment of EOC. High FOXA1 expression may contribute to the resistance of many anticancer drugs.

**Conclusions:**

Our results predict and validate the function of FOXA1 in promoting EMT and the progression of disease in EOC. Targeting FOXA1 may improve the sensitivity of EOC treatment.

## 1. Background

Ovarian cancer is a fatal gynecological cancer [1–3]. Ovarian cancer is often identified in the advanced phase because it lacks early-stage symptoms. Even the symptoms in the advanced stage, including abdominal pain and swelling, loss of appetite, and frequent urination, can be overlooked. Germline mutations of the BRCA1 and BRCA2 genes confer a high life-time risk of ovarian cancer [4, 5]. Moreover, ovarian cancer is genetically and phenotypically heterogeneous without reliable and effective biomarkers for diagnosis and prognosis, which also makes it challenging to detect ovarian cancer in the early phase [6]. The lack of effective serum biomarkers for ovarian cancer as well as studies aimed at identifying novel effective biomarkers, including miRNAs, lncRNA, and circulating tumor DNA, also brings difficulties to the early diagnosis and treatment of ovarian cancer [7–9]. The 5-year survival rate of patients with advanced ovarian cancer was increased with the development of surgery techniques and combined therapies of chemotherapy drugs and monoclonal antibody drugs. However, the long-term survival rate still remains disappointing [10, 11]. Further investigating the mechanisms of the occurrence and progression of ovarian cancer is fundamental for diagnosis in the early stage and therapy of ovarian cancers.

Epithelial-mesenchymal transition (EMT) has long been considered related to tumorigenesis, in which the differentiated epithelial cells (E) shift towards mesenchymal-like (M) properties by losing the apical-basal polarity and cell-cell adhesion and gaining the properties of stemness, migration, and proliferation. During the development of carcinoma, EMT leads to augmented capability to invade, migrate, and resist to chemotherapy drugs and metastasize in neoplastic cells [12]. In the occurrence of EMT, E-cadherin is downregulated because E-cadherin-mediated intercellular tight junctions are destroyed [13]. Moreover, EMT is often characterized by a gained motility and changed morphology of the cells as well as the compositional alteration of cytoskeleton filaments [14]. An overexpressed intermediate filament protein, vimentin, is commonly observed in various epithelial cancers, including EOC. Thereby, mesenchymal markers are often upregulated in EMT, including matrix metallopeptidases (MMPs), fibronectin, vimentin, and N-cadherin, while epithelial markers are downregulated, such as claudins and E-cadherin [15].

EMT, a complicated biological process, plays a key role in an array of physiological and pathological events, including tumor progression [16, 17], cancer cell invasion, embryogenesis [18, 19], wound healing [20], fibrosis [21, 22], therapy resistance [23], and inflammation [24]. There are various signaling pathways that have participated in mediating EMT, in which TGF-*β* pathway is considered closely related to the invasion-metastasis cascade of EOC by enhancing the stemness of cancer stem cells and promoting the invasiveness and migration of tumor cells [25].

Recently, the transcription factor forkhead-box A1 (FOXA1) was reported to be a meaningful EMT reporter [26]. The overexpression of FOXA1 was also reported in several cancer types, and thus, it serves as a significant indicator of the poor overall survival (OS) of patients. FOXA1 belongs to FOX family, participating in the development of many endoderm-derived organs mainly through mediating the nuclear steroid receptor signaling [27, 28]. It has been studied in various human cancers, including thyroid cancer [29], breast cancer [30], lung cancer [31], gastric cancer [32], and prostate cancer [33], and suggested to be closely associated with the malignancy and clinicopathological characteristics of tumors.

Yet, there is still insufficient work to illustrate the mechanism regarding the promotive effects of FOXA1 on EMT and further tumor microenvironment (TME) in EOC. In the study, we first explored the pan-cancer features of FOXA1. EMT and immune-related pathways were closely correlated with FOXA1 in EOC. In the aspect of experimental verification, we compared the expression in different EOC cell lines. Cell proliferation and invasion were examined following siRNA-FOXA1 transfection to demonstrate the role of FOXA1 in altering cell towards EMT-like characteristics and in promoting tumorigenesis in EOC. More importantly, the interaction between FOXA1 and connective tissue growth factor (CTGF) is of our great interest, as the preliminary study reported that FOXA1 may affect the expression of CTGF [34]. Thereby, it is hypothesized that the transcription factor, FOXA1, may directly regulate the transcription of CTGF by interacting with its promoter region.

CTGF is implicated in multiple complexed signaling networks and involved in various biological processes, including cell adhesion, migration, proliferation, angiogenesis, and extracellular matrix synthesis [35]. Although the pleiotropic biofunctions of CTGF remain to be elucidated, researchers have noted its association with EMT and cancer malignancies. CTGF is preferentially expressed in aggressive neoplasms [36]. The induction of CTGF-induced EMT is mainly mediated via the TGF-*β* signaling. Also, TGF-*β* also affects the activity of many other pathways that can trigger EMT, such as Notch, Wnt, and integrin pathways. Based on the above facts, the amplification of FOXA1 and FOXA1-induced CTGF is expected to play a pivotal role in the progression of ovarian cancer by propelling the occurrence of EMT. To further examine the association between the development of EMT and FOXA1-CTGF-TGF-*β* signaling pathway in the ovarian cancer cells, we studied the effects of lithium chloride, an inhibitor of TGF-*β* pathway, on the cells [37].

## 2. Methods

### 2.1. Data Collection and Analysis

The expression and clinical data of The Cancer Genome Atlas (TCGA) and Genotype-Tissue Expression (GTEx) were downloaded from the UCSC Xena database (https://xenabrowser.net/datapages/). For FOXA1, the DNA copy number and methylation information were obtained from the cBioPortal database (https://www.cbioportal.org/). The survival analysis of FOXA1 in EOC database from GSE26193, GSE26712, and GSE63885 was performed using PrognoScan database. The expression of FOXA1 in single cell sequencing data of OV was evaluated using TISCH database (http://tisch.comp-genomics.org/home/). The immunotherapy dataset GSE135222 was downloaded from GEO database. The immunotherapy dataset Checkmate cohort was obtained from the supplementary materials of the published paper [38]. All patients in these cohorts were enrolled in this study.

### 2.2. Gene Set Enrichment Analysis (GSEA)

Correlation analyses between FOXA1 and other genes were performed using data from TCGA-OV cohort, and Pearson's correlation coefficient was calculated. GSEA was conducted using the R package “clusterProfiler” based on Gene Ontology (GO), Kyoto Encyclopedia of Genes and Genomes (KEGG), and Reactome pathway databases.

### 2.3. Correlation Analysis of FOXA1 and Drug Response

We downloaded half-inhibitory concentration (IC_50_) values of 192 anticancer drugs and FOXA1 expression profiles of 809 cell lines from the Genomics of Drug Sensitivity in Cancer database (GDSC: https://www.cancerrxgene.org/) and analyzed the Spearman's correlation between FOXA1 expression and IC_50_ values of anticancer drugs.

### 2.4. Cell Culture

OVCAR3, A2780, 3AO, and SKOV-3 (ovarian tumor cell lines) and IOSE80 (normal ovarian cell line) were purchased from ATCC of the United States. OVCAR3, 3AO, and A2780 were cultured in RPMI 1640 medium supplemented with penicillin (100 U/mL), streptomycin (100 mg/L), and 10% *v*/*v* fetal bovine serum (FBS). IOSE80 and SKOV3 were cultured in DMEM and McCoy's 5A medium containing penicillin (100 U/mL), streptomycin (100 mg/L), and 10% FBS, respectively. The cells were cultured at 37°C and 5% CO_2_. (The culture medium and supplements were purchased from Invitrogen.)

### 2.5. Silencing FOXA1 by Using Small Interfering RNA (siRNA)

FOXA1 siRNA and its negative control (NC) siRNA were purchased from Shanghai Sangon Co., Ltd. (Shanghai, China). Three siRNA sequences were as follows: siRNA#1 (5′-GCGACUGGAACAGCUACUATT-3′; 5′-UAGUAGCUGUUCCAGUCGCTT-3′), siRNA#2 (5′-CCACUCGCUGUCCUUCAAUTT-3′; 5′-AUUGAAGGACAGCGAGUGGTT-3′, and siRNA#3 (5′-GCACUGCAAUACUCGCCUUTT-3′; 5′-AAGGCGAGUAUUGCAGUGCTT-3′). Transfection of siRNA was performed by using Lipofectamine™ 3000 Transfection Reagent (Thermo Fisher). After transfection for 72 h, the cells were collected to evaluate the knockdown efficiency of FOXA1 in OVCAR-3 cells (*n* = 6) by western blot and qPCR. The siRNA with the best efficiency was used in subsequent experiments.

### 2.6. CCK-8 Viability Assay

OVCAR3 cells transfected with siRNA-FoxA1 or siRNA-NC were cultured in a 96-well plate with 1 × 10^4^ cells/well and 6 parallel wells in each group. The cells were cultured for 24, 48, and 72 h, respectively, before CCK-8 assay. CCK-8 solution was incubated with cells at 37°C for 1-2 h. The cell survival rate was detected at a wavelength of 450 nm.

### 2.7. Transwell Assay

Biocoat™ Matrigel® Invasion Chamber was rehydrated for 2 h at 37°C and 5% CO_2_ using a filter with 8.0 *μ*m pore size (Corning, USA). After transfection with siRNA, the cells were collected and suspended (1 × 10^6^ cells/mL) in serum-free culture medium. 200 *μ*L of the cells in serum-free culture medium was added to the upper compartment, and 600 *μ*L RPMI 1640 containing 10% FBS was added to the lower compartment. After 24 h of incubation at 37°C under 5% CO_2_, the nonmigrating cells were removed and culture medium was discarded. Filters were gently rinsed with PBS, and migrated cells were fixed with 4% *w*/*v* formaldehyde for 15 min. 0.1% crystal violet staining solution was used to stain the cells for 30 min. The upper, middle, and lower left and right fields were observed under the optical microscope (magnification ×100) for cell counts for each assay. Cell migration was quantified with ImageJ software. Each group included three independently performed Transwell assays.

### 2.8. Immunofluorescence Staining

The immunofluorescence staining was performed on cells grown on 22 × 22 mm coverslips. OVCAR-3 cells (3 × 10^4^/well) were grown in a 12-well plate and cultured in the 37°C incubator overnight. At 72 h after the transfection with 80 nM siRNA-FOXA1, 4% *w*/*v* paraformaldehyde was used for fixation for 30 min. 0.5% *v*/*v* Triton X-100 was used in permeabilization for 5 min and 10% normal donkey serum for blocking for 1 h at room temperature. Then, the cells were incubated at 4°C overnight with the primary antibodies: mouse anti-E-cadherin (1 : 500, Abcam, USA) and rabbit anti-vimentin (1 : 500, Abcam, USA). After being washed by PBS for 3 times, secondary antibodies listed as follows were used for incubation for 1 h: Alexa Fluor® 488 donkey anti-mouse IgG or anti-rabbit (1 : 200, Jackson ImmunoResearch, USA). The cells were finally counterstained with DAPI (Beyotime, China) for 5 min, and the coverslips were mounted by using 10 *μ*L of FluroGuard antifade solution (Bio-Rad, USA). Images were taken using a confocal microscope (Leica, Germany).

### 2.9. Quantitative Real-Time PCR (qPCR)

Cells or tissues were collected, and the RNAs were obtained by using TRIzol® Plus RNA Purification Kit (Thermo Fisher, Carlsbad, CA, USA) following the protocol. SuperScript™ III First-Strand Synthesis SuperMix for qRT-PCR (Thermo Fisher, Carlsbad, CA, USA) was used to synthesize cDNA. Real-time PCR was carried out by applying PowerUp™ SYBR™ Green Master Mix (Applied Biosystems, Carlsbad, CA, USA). The cycling program was set as a follows: 95°C, 2 min; 40 cycles of amplification (95°C, 15 s; 60°C, 1 min). The sequences of the primers used in this experiment were as follows: FOXA1, 5′-GCATACGAA CAGGCACTGCAATACT-3′ (forward) and 5′-GTGTTTAGGACGGGTCTGGAATA-3′ (reverse), and GAPDH, 5′-CCATGACAACTTTGGTATCGTGGAA-3′ (forward) and 5′-GGCCATCACGCCACAGTTTC-3′ (reverse). GAPDH was used as internal control for normalization. The relative expression of the target genes was evaluated by the 2^-∆∆Ct^ method.

### 2.10. Western Blot

Total cytoplasmic and nuclear proteins from OVCAR-3 cells were extracted with RIPA buffer (Thermo Fisher, USA). NE-PERTM Nuclear and Cytoplasmic Extraction Kit (Thermo Fisher, USA) was added with protease and phosphatase inhibitor cocktail (Thermo Fisher, USA). BCA protein assay kit (Beyotime Biotechnology, China) was used for quantification. Protein (30 *μ*g) was loaded onto SDS-PAGE gel and transferred to a Hybond-P PVDF membrane (GE Healthcare, USA). 5% fat-free milk in TBST (Tris-buffered saline with 0.1% Tween 20) was used for blocking. The following antibodies were incubated with the membranes at 4°C overnight: mouse anti-E-cadherin (1 : 2000, CST: 3195, USA), rabbit anti-vimentin (1 : 2000, CST: 5741, USA), rabbit anti-Snail (1 : 1000, Abcam: ab216347, USA), and actin (1 : 5000, Abcam: ab8227, USA) as an internal control. Protein expression was visualized on X-ray films using the HRP-conjugated goat anti-mouse or anti-rabbit secondary antibodies (1 : 5000, Thermo Fisher, USA) and SuperSignal West Dura Extended Duration Substrate (Thermo Fisher, USA). Band intensities were quantitated using Image Pro Plus 6.0 software. The results were presented as the density ratio of the target protein band to the internal control.

### 2.11. Construction of the CTGF Promoter Luciferase Reporter Plasmids

The binding site of FOXA1 with CTGF was predicted on LASAGNA-search. Sequences of the wild-type CTGF promoter (-500 to -1) and mutant CTGF promoter (-399 to -390, CAGGGCAAAC to CACCGCTTAC) with recognition sites specific for the enzymes KpnI/XhoI were manufactured by Sangon Biotech (Shanghai). FOXA1 CDS flanked with BamHI/EcoRI was cloned as well. Luciferase reporter plasmids, pGL3-Basic-CTGF-w (wild type) and pGL3-Basic-CTGF-m (mutant), were constructed by having the primers flanked with KpnI/Xho cloned into the KpnI/Xho sites of pGL3-Basic vector (Promega). The FOXA1 overexpression plasmid was created by cloning the FOXA1 CDS sequence with BamHI/EcoRI into pcDNA3.1 (Invitrogen). pRL-TK vector (Promega), the Renilla luciferase plasmid, was adopted as an internal control reporter vector.

### 2.12. Dual-Luciferase Reporter Assays

OVCAR3 cells were cotransfected with a combination of plasmids comprising of either a mutant or a wild-type CTGF reporter gene plasmid, a pRL-TK Renilla luciferase reporter plasmid, and a FOXA1 overexpression plasmid. Thus, the cells were transfected with the combination of plasmids as following, respectively: pGL3-Basic/pcDNA3.1-FOXA1/pRL-TK, or pGL3-Basic-CTGF-w/pcDNA3.1-FOXA1/pRL-TK, or pGL3-Basic-CTGF-m/pcDNA3.1-FOXA1/pRL-TK group. Activities of firefly Renilla luciferases were measured 48 h after transfection according to the dual-luciferase reporter assay system, with six replicas in each group (Promega).

### 2.13. Effects of FOXA1 Silencing on CTGF/TGF-*β* Pathway and EMT-Associated Markers

OVCAR3 cells were transfected with siRNA-NC or siRNA-FOXA1, which was followed by replacing with the serum-free culture medium after 12 h. The TGF-*β*1 groups were stimulated with 10 ng/mL TGF-*β*1 accordingly (R&D systems). The cells were collected after 48 h for western blot analysis for FOXA1, CTGF, MMP-2, E-cadherin, and Snail using rabbit-anti-CTGF antibody (1 : 1000, Abcam, USA) and rabbit-anti-MMP2 antibody (1: 500, Abcam, USA).

### 2.14. Lithium Chloride Treatment

The cells were grouped as follows: control, siRNA-NC-transfected, siRNA-NC-transfected and LiCl treatment, siRNA-FOXA1-transfected, siRNA-FOXA1-transfected, and LiCl treatment. A final concentration of 10 mM LiCl was added to the cells after transfection of siRNA-NC or siRNA-FOXA1 for 8 h.

### 2.15. Statistical Analysis

SPSS 17.0 software (SPSS Inc., Chicago, IL, USA) was used for statistical analysis. The results were expressed as average value ± standard deviation (SD). The paired, two-tailed Student's *t*-test was used to compare the results between the two groups. Two-sided *p* value less than 0.05 was regarded as statistically significant (^∗^*p* < 0.05, ^∗∗^*p* < 0.01, ^∗∗∗^*p* < 0.001, and ^∗∗∗∗^*p* < 0.0001).

## 3. Results

### 3.1. Pan-Cancer Expression of FOXA1

**Figure 1:**
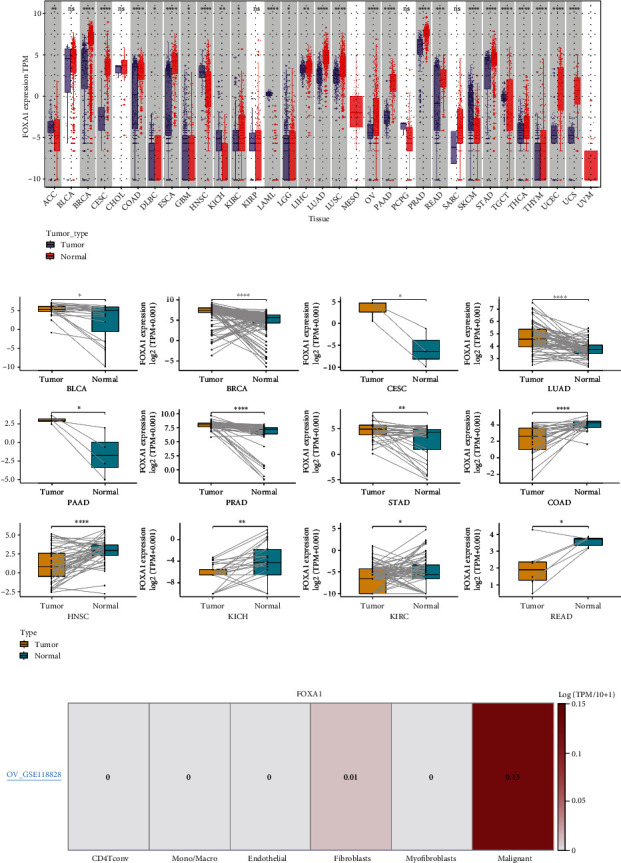
Pan-cancer expression. (a) Pan-cancer expression of FOXA1. (b) The expression of FOXA1 in paired tumor and adjacent normal tissues in indicated tumor types from TCGA cohort. (c) The expression of FOXA1 in indicated cells.

**Figure 2:**
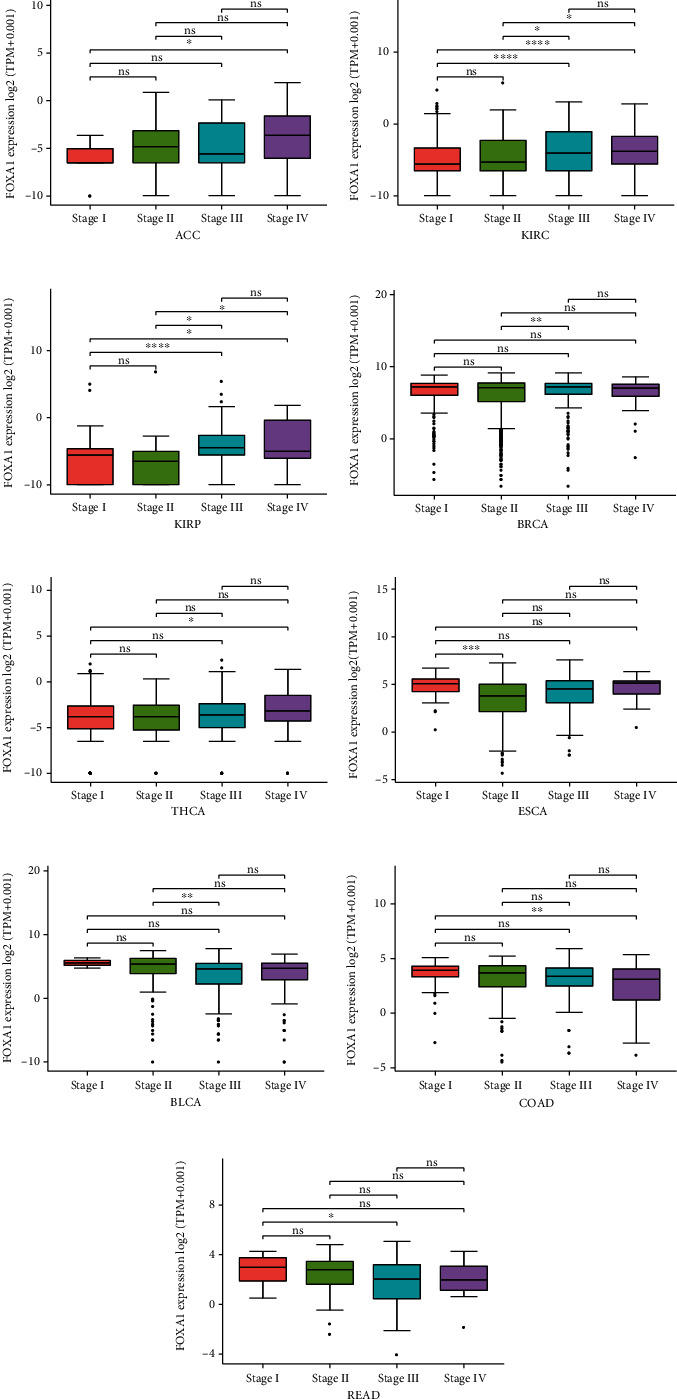
FOXA1 expression in different tumor stages. (a–i) The FOXA1 expression in different tumor stages in indicated tumor stages from TCGA cohort.

We first evaluate the pan-cancer expression of FOXA1. The results revealed that FOXA1 was highly expressed in 19 tumor types, including BRCA, CESC, COAD, DLBC, ESCA, KIRC, LGG, LIHC, LUAD, LUSC, OV, PAAD, PRAD, READ, STAD, THCA, THYM, UCEC, and UCS. In comparison, low FOXA1 expression was observed in ACC, GBM, HNSC, KICH, LAML, SKCM, and TGCT (Figure 1(a)). In paired tumor and adjacent normal tissues, FOXA1 was overexpressed in BLCA, BRCA, CESC, LUAD, PAAD, PRAD, and STAD while low expressed in COAD, HNSC, KICH, KIRC, and READ (Figure 1(b)). For the expression of FOXA1 in single cell in OV, we found that FOXA1 was mainly expressed in malignant tumor cells (Figure 1(c)). In addition, we also observed that FOXA1 expression was higher in relative worse tumor stages in ACC, KIRC, KIRP, BRCA, and THCA (Figures 2(a)–2(e)) while lower in ESCA, BLCA, COAD, and READ (Figures 2(f)–2(i)).

**Figure 3:**
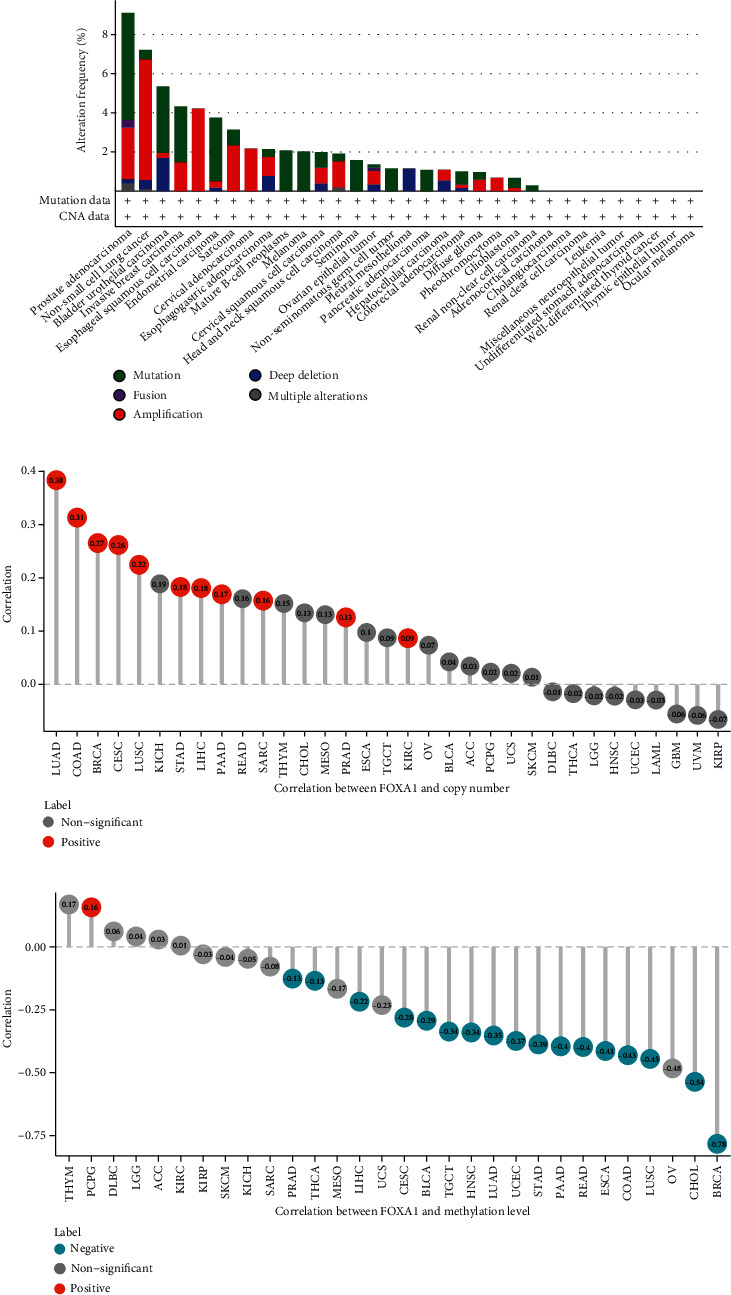
Gene alteration of FOXA1. (a) The genetic alteration of FOXA1 in TCGA pan-cancer. (b) The correlation between FOXA1 expression and copy number in TCGA pan-cancer. (c) The correlation between FOXA1 expression and methylation level in TCGA pan-cancer.

Genetic and epigenetic alterations induce changes in gene expression. We explored genetic alterations in FOXA1 using cBioPortal and observed that patients in prostate adenocarcinoma and non-small-cell lung cancer have high genetic alterations of FOXA1 (Figure 3(a)). In EOC patients, the frequency genetic and epigenetic alterations were low (less than 2%), in which the “amplification” accounts for the largest proportion. The copy number values were positively correlated with FOXA1 expression, and the methylation levels of the FOXA1 promoter were negatively correlated with FOXA1 expression in most tumor types, while not in EOC (Figures 3(b) and 3(c)). These results indicated that FOXA1 was highly expressed in EOC and other tumor types. The mRNA expression of FOXA1 was not significantly affected by genetic and epigenetic alterations.

### 3.2. The Prognostic Value of FOXA1

**Figure 4:**
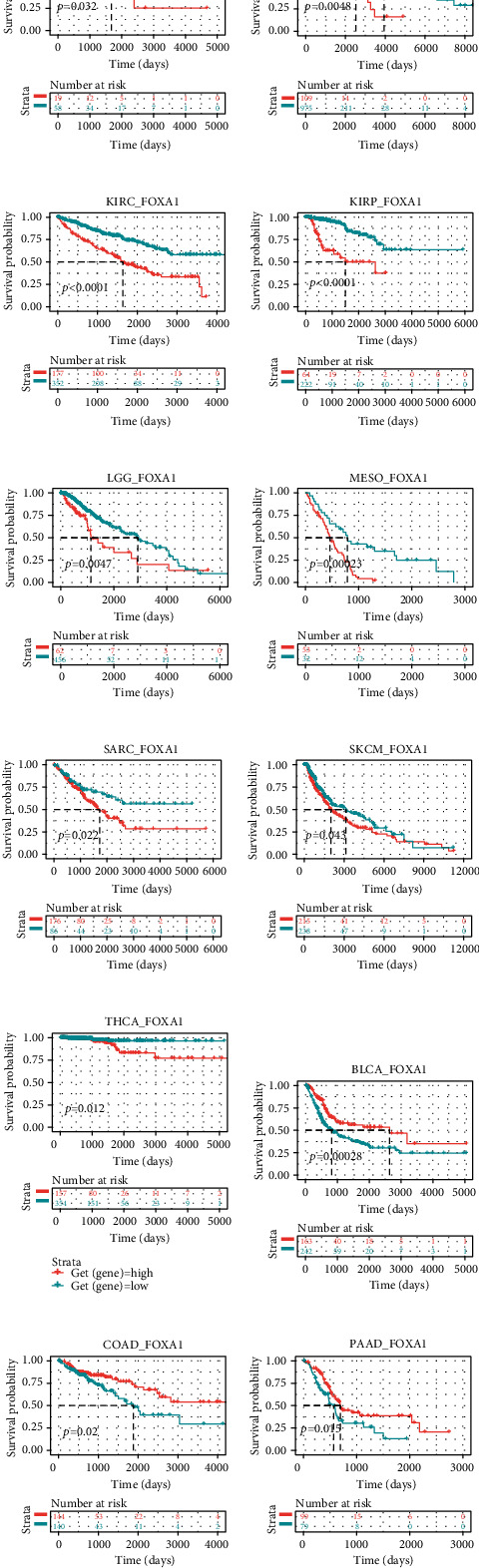
Prognostic value of FOXA1. (a–l) The Kaplan-Meier analyses of FOXA1 in indicated tumor types. The optimum cutoff value of FOXA1 in each tumor type was set.

**Figure 5:**
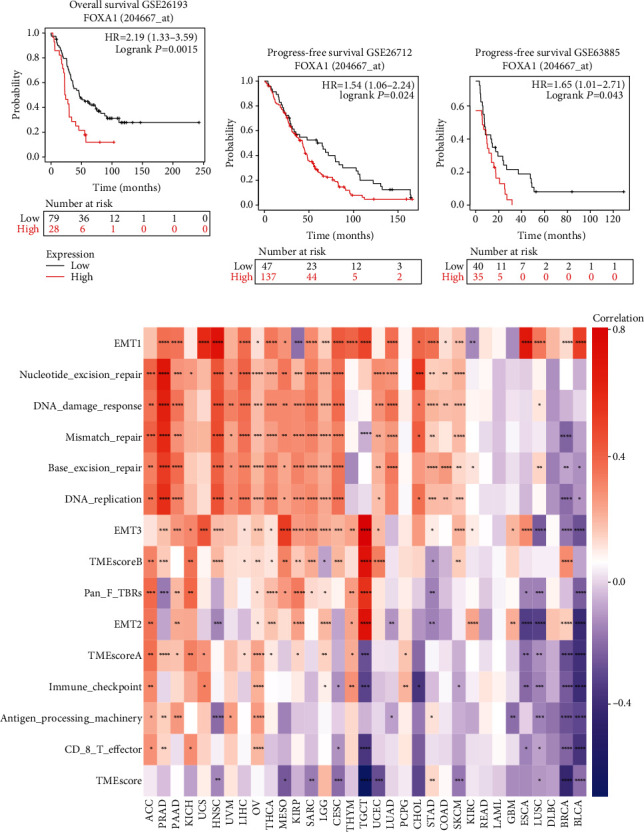
TME analysis. (a–c) The Kaplan-Meier analyses of FOXA1 in EOC datasets, including GSE26193 (a), GSE26712 (b), and GSE63885 (c). The optimum cutoff value of FOXA1 in each dataset was set. (d) The correlation between TME-related signature scores and FOXA1 expression.

To assess the prognostic value of FOXA1, we performed the Kaplan-Meier survival analysis in pan-cancer. The Kaplan-Meier survival analysis revealed that high FOXA1 expression predicted worse overall survival of patients with ACC, BRCA, KIRC, KIRP, LGG, MESO, SARC, SKCM, and THCA (Figures 4(a)–4(i)) while better survival of patients with BLCA, COAD, and PAAD (Figures 4(j)–4(l)). For data of EOC from GEO database, we found that high FOXA1 predicted poorer survival status of EOC patients in GSE26193, GSE26712, and GSE63885 (Figures 5(a)–5(c)). To explore the association between FOXA1 expression and TME, we downloaded signature gene sets of TME from published articles and calculated signature scores according to the method described previously [39]. The results revealed that EMT-related pathways was positively correlated with FOXA1 in pan-cancer. These finding suggested that FOXA1 was a prognostic biomarker in EOC. FOXA1 may affect the EMT progression.

### 3.3. Expressions of FOXA1 in EOC Cell Lines

**Figure 6:**
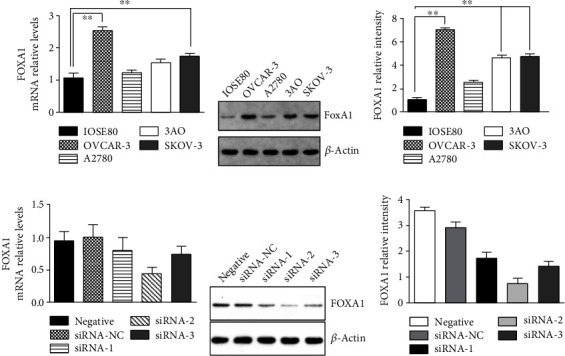
FOXA1 expression was upregulated in ovarian cancer cells. (a–c) FOXA1 expression was assessed by qRT-PCR (a) and western blot (b) in IOSE80 (human ovarian epithelial cell line from normal tissues) and OVCAR-3/A2780/3AO/SKOV-3 cells (human ovarian cancer cell lines). Relative mRNA and protein levels of FOXA1 were quantified in (a) and (c); *N* = 3, two-way ANOVA. (d, e) FOXA1 knockdown via transient transfection with siRNA-2 in OVCAR-3 cell was confirmed by qRT-PCR (d) and western blot (e, f). siRNA-NC-transfected cells were set as the control. The experiments were repeated independently for 3 times.

The RNA materials from the cancer and normal cells lines were extracted for reverse transcription and RT-PCR assay (Figure 6(a)). The relative mRNA level of FOXA1 is significantly upregulated in the OVCAR3 cell line compared to the rest. Western blot found that the protein level of FOXA1 was significantly upregulated in ovarian cancer cells compared with the normal cell line IOSE80 (Figures 6(b) and 6(c)). Among all the ovarian cancer cell lines tested (i.e., OVCAR-3/A2780/3AO/SKOV-3), both transcriptional and translational levels of FOXA1 were highest in OVCAR3, followed by 3AO and SKOV-3, and then A2780.

### 3.4. FOXA1 Expression Was Significantly Inhibited by siRNA Silencing

Three siRNAs, siRNA-1, siRNA-2, and siRNA-3, specific for different sites of FOXA1 gene were designed for silencing FOXA1 in OVCAR3 by transient transfection. At the same time, the potency of the three siRNA was compared. The mRNA and protein expressions of FOXA1 were remarkably suppressed with siRNA-2 transfection compared to the other groups (Figures 6(d)–6(f)). Thus, siRNA-2 was employed as the most effective interference siRNA in the following experiments.

### 3.5. FOXA1 Silencing Markedly Attenuated Cell Proliferation and Invasion

**Figure 7:**
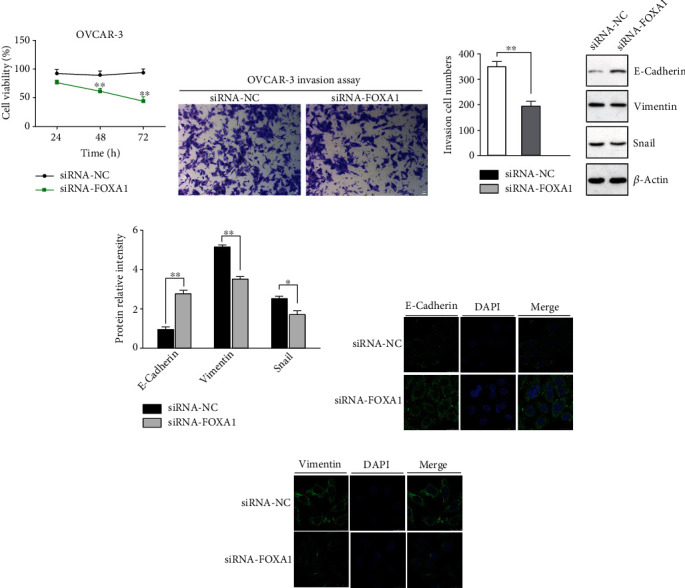
Effects of FOXA1 silencing on OVCAR-3 cell proliferation and invasion. (a) The proliferation was measured by CCK8 assay at 24, 48, and 72 h, respectively. The results were obtained from the 6 replicates in each group and presented as average value ± SD. (b) The invasiveness of OVCAR-3 cells was detected by the Transwell migration assay. Representative images were selected (magnification ×100). (c) The number of invaded cells were quantified and obtained from three independent experiments. (d) Protein levels of E-cadherin, vimentin, and Snail were measured by western blot after 48 h following siRNA transfection. (e) Quantifications of representative blots were demonstrated in bar graphs. Protein levels of E-cadherin, vimentin, and Snail in the FOXA1-silenced group (siRNA-FOXA1) were compared to those in the control group (siRNA-NC) and presented in the form of average ± SD. (f, g) Representative immunofluorescence staining for E-cadherin (f) and vimentin (g) in the siRNA-NC- or siRNA-FOXA1-transfected OVCAR3 cells (original magnification ×630).

The proliferative and invasive capabilities of FOXA1 knockdown cells were examined by the CCK8 and Transwell assays, respectively. The proliferation of cells was significantly suppressed following the transfection of siRNA-FOXA1 compared with siRNA-NC (Figure 7(a)). The results from Transwell assay indicated that the invasiveness was also significantly inhibited due to the absence of FOXA1 in siRNA-FOXA1 OVCAR-3 cells (Figures 7(b) and 7(c)).

### 3.6. FOXA1 Silencing Inhibited EMT

OVCAR3 cells were transfected with siRNA-FOXA1 and siRNA-NC, respectively. EMT-associated markers in OVCAR3 cells were detected by western blot and immunofluorescence staining. As shown in Figures 7(d)–7(g), the results indicated that FOXA1 silencing inhibited EMT as shown by the remarkably increased expression of E-cadherin and decreased levels of vimentin (*p* < 0.01). It was indicated that FOXA1 might serve as an activator in OVCAR3 cells via promoting EMT. These results confirmed that FOXA1 could regulate the EMT process of EOC cells.

### 3.7. FOXA1 Regulated CTGF Expression by Binding to Its Promoter Region

**Figure 8:**
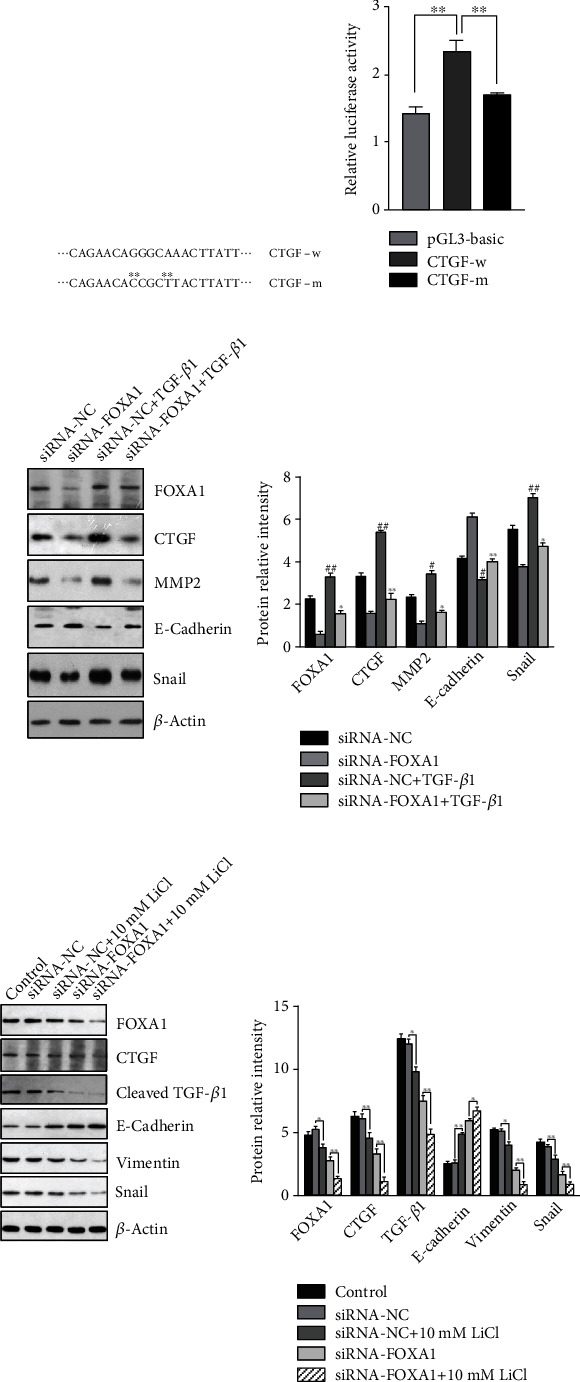
The suppressive effects of FOXA1 silencing on EMT through TGF-beta 1 signaling pathway. (a) Predicted binding site of FOXA1 at the wild-type CTGF promoter region and the mutant CTGF promoter (-399 to -390) was present. (b) Relative luciferase activity was presented as per the ratio of the intensity of firefly luciferase to that of Renilla. (c) Western blot analysis on protein expression levels of FOXA1, CTGF, MMP-2, E-cadherin, and Snail in the siRNA-NC-, or the siRNA-FOXA1-transfected cells treated with or without TGF-*β*1. Quantifications of protein expressions was represented as mean ± standard deviation (SD) of the results from six independent replicates in each group. (d) Expressions of FOXA1, CTGF, cleaved TGF-beta 1, and EMT-associated markers including E-cadherin, vimentin, and Snail were analyzed by western blot after 48 h after the transfected cells treated with or without lithium chloride. Quantification of the representative blots was represented as mean ± standard deviation (SD) of the results from three independent experiment in bar graphs.

To explore the relation between FOXA1 and CTGF, OVCAR3 was transfected with either a wild-type CTGF promoter or a mutant CTGF promoter; the predicted binding site and the mutated sequence were defined as in Figure 8(a). The results from dual-luciferase reporter assays showed that, comparing to the pGL3-Basic control group, the activity of pGL3-Basic-CTGF-w luciferase was significantly enhanced (*p* < 0.01), while the activity of pGL3-Basic-CTGF-m higher than that of pGL3-Basic-CTGF-w was significantly downregulated (*p* < 0.01) (Figure 8(b)). It suggests that FOXA1 initiates transcription of CTGF by binding to its promoter.

### 3.8. FOXA1-Mediated EMT Was Dependent on the Activation of CTGF/TGF-*β* Pathway

In order to confirm that the molecular mechanism of FOXA1-induced EMT relies on activation of the CTGF/TGF-*β* signaling pathway, FOXA1 of OVCAR-3 cells was silenced followed by a stimulation with TGF-*β*1 in vitro. Western blot analysis showed that both CTGF and FOXA1 expressions were considerably elevated in the TGF-*β*1 stimulated groups (siRNA-NC+TGF-*β*1 and siRNA-FOXA1+TGF-*β*1) compared to the nonstimulated groups (siRNA-NC and siRNA-FOXA1) (Figure 8(c)). When comparing the groups siRNA-NC vs. siRNA-FOXA1 and siRNA-NC+TGF-*β*1 vs. siRNA-FOXA1+TGF-*β*1, it is found that the expressions of CTGF and FOXA1 were significantly suppressed due to FOXA1 ablation. In addition, the EMT-associated proteins, MMP2, and Snail were significantly upregulated following the treatment of exogenous TGF-*β*1 (*p* < 0.01), whereas their expressions were considerably reduced (*p* < 0.05 and *p* < 0.01) following FOXA1 ablation. Congruently, the cell-cell junction indicator protein, E-cadherin, showed a prominent upregulation in FOXA1-silenced cells (*p* < 0.01), and a downregulation in TGF-*β*1 stimulated cells (*p* < 0.01). Here, it shows that interference with FOXA1 downregulates the expression of CTGF, thus inhibiting the activation of CTGF/TGF-*β* pathway in OVCAR3 cells, which in turn attenuates the occurrence and development of EMT that is mediated by the TGF-*β* signaling pathway.

### 3.9. The Inhibitory Effect of LiCl on FOXA1 and EMT in OVCAR3 Cells

To examine the inhibitory effects of LiCl on the FOXA1-CTGF-TGF-*β* pathway, OVCAR3 cells were treated accordingly as per the description of the groups, namely, the siRNA-FOXA1+10 mM LiCl, siRNA-NC+10 mM LiCl, siRNA-FOXA1, and siRNA-NC groups. The protein expression levels of FOXA1, vimentin, E-cadherin, and Snail in the treated cells were detected by western blot assays. In the single treatment group with either LiCl (siRNA-NC+10 mM LiCl) or FOXA1 knockdown (siRNA-FOXA1), expressions of FOXA1, CTGF, cleaved-TGF-beta, and EMT-associated markers were all downregulated (Figure 8(d)). The potency of suppression was strongest in the combination treatment group (siRNA-FOXA1+LiCl). The combination treatment of FOXA1 knockdown and administration of lithium chloride exerts a more robust inhibitory effect on the EMT-associated proteins, compared to the respective single treatment. The epithelial marker, E-cadherin, was upregulated in the treatment groups, indicating a recovery of the cell-cell adhesive junctions. Our data show that the LiCl treatment is able to suppress the FOXA1-CTGF-TGF-*β* pathway and, therefore, inhibit EMT in OVCAR3 cells. Equally important, silenced FOXA1 and LiCl treatment could have worked collaboratively to augment the inhibitory impact on EMT features.

### 3.10. The Correlation between FOXA1 and Immunosuppressive Microenvironment

**Figure 9:**
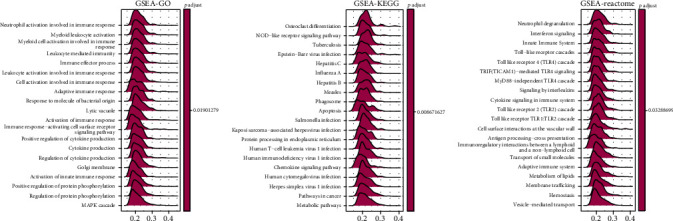
GSEA or FOXA1. (a–c) The GSEA results of FOXA1 in TCGA-OV cohort, including GSEA-GO (a), GSEA-KEGG (b), and GSEA-Reactome (c).

**Figure 10:**
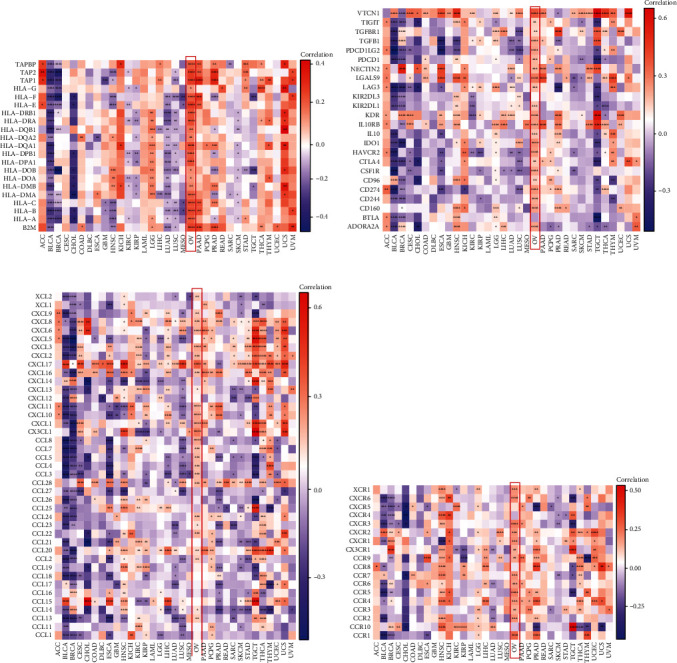
The correlation analysis. (a) The correlation between MHC genes and FOXA1. (b) The correlation between immunosuppressive genes and FOXA1. (c) The correlation between immune activating genes and FOXA1. (d) The correlation between chemokine receptors and FOXA1.

**Figure 11:**
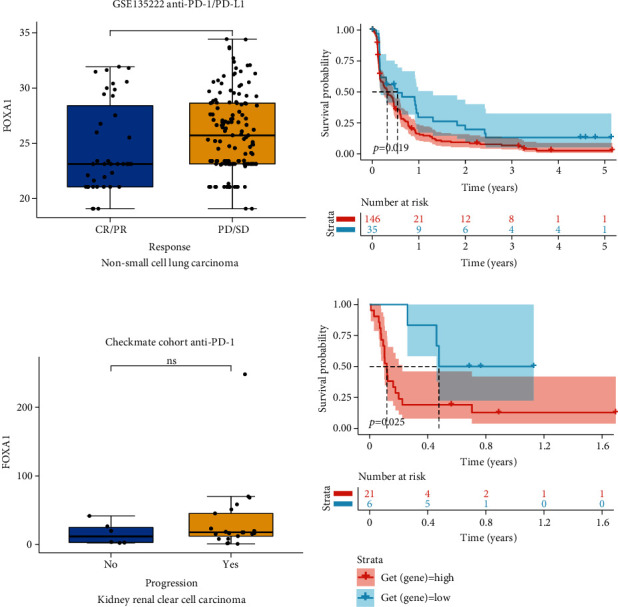
Effect of FOXA1 on the efficacy of immunotherapy. (a) The FOXA1 expression in indicated groups in GSE135222. (b) The Kaplan-Meier curve of FOXA1 in GSE135222. (c) The FOXA1 expression in indicated groups in checkmate cohort. (d) The Kaplan-Meier curve of FOXA1 in checkmate cohort.

We further performed the GSEA of FOXA1 in TCGA-OV cohort based on GO, KEGG, and Reactome databases. The results revealed that immune-related pathways were commonly enriched (Figures 9(a)–9(c)). Through the correlation analyses between FOXA1 and MHC genes (Figure 10(a)), immunosuppressive genes (Figure 10(b)), immune activating genes (Figure 10(c)), and chemokine receptors (Figure 10(d)), we found that FOXA1 was closely correlated with immune regulatory genes, indicating a pivotal role of FOXA1 in tumor immunomodulatory function, especially in TCGA-OV cohort. Since the immunosuppressive microenvironment is not conducive to the efficacy of immune checkpoint inhibitors, we speculated that patients with high expression of FOXA1 are resistant to immunotherapy. Through our analysis, we found that FOXA1 expression was higher in PD/SD (progressive disease/stable disease) group than that in CR/PC (partial response/complete response) group in GSE135222 (Figure 11(a)). In addition, high expression of FOXA1 predicted poorer survival status of patients undergoing immunotherapy (Figure 11(b)). The same phenomenon was observed in Checkmate immunotherapy cohort (Figures 11(c) and 11(d)).

### 3.11. The Correlation between FOXA1 and Resistant of Anticancer Drugs

**Figure 12:**
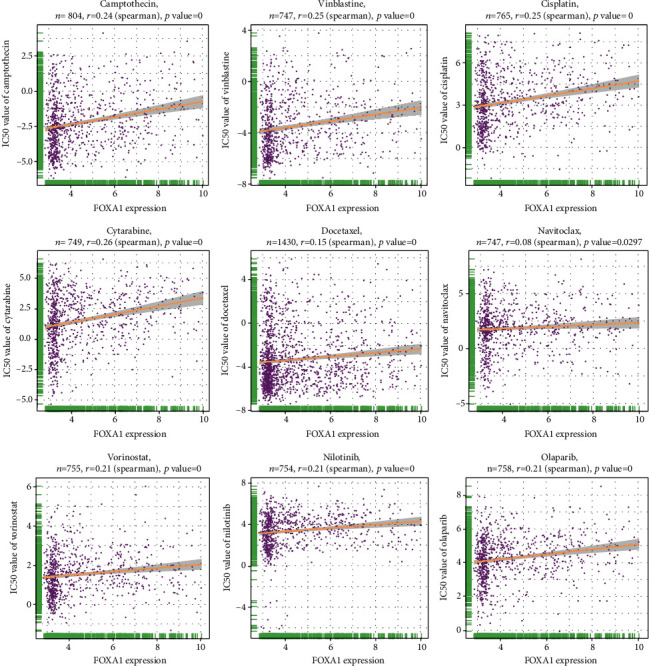
The correlation analysis between FOXA1 and IC_50_ values of indicated anticancer drugs.

At last, we conducted the correlation analyses between FOXA1 expression and IC_50_ values of anticancer drugs. We found that the expression of FOXA1 positively correlated IC_50_ values of most anticancer drugs, such as camptothecin, vinblastine, and cisplatin (Figure 12). These results indicated that EOC patients with high expression of FOXA1 may be resistant to most anticancer drug treatments.

## 4. Discussion

In our research, we investigated the potential role of the FOXA1, a transcription factor, in pan-cancer and EOC and its underlying molecular mechanism on promoting EMT. In pan-cancer research, we found that FOXA1 was highly expressed in most tumor types, including EOC. High expression of FOXA1 predicted poorer survival of patients with EOC. For the expression of FOXA1 in single cell in OV, we found that FOXA1 was mainly expressed in malignant tumor cells, indicating that FOXA1 mainly plays its function in tumor cells. By analyzing the frequency genetic and epigenetic alterations of FOXA, we found that the mRNA expression of FOXA1 was not significantly affected by genetic and epigenetic alterations in EOC. In the experimental verification, FOXA1 expression levels in four ovarian cancer cell lines were all higher than those in normal ovarian cells. The overexpression was most prominent in OVCAR-3 with the most malignant characteristics, implying that FOXA1 had an evitable role in the development of EOC. Preliminary studies demonstrated that FOXA1 silencing could effectively hinder the invasion and proliferation of certain tumor cells, for example, lung adenocarcinoma A549 cells [12] and lung squamous cell carcinoma cells [40]. These results were consistent with what we found in our research. In FOXA1 knockdown OVCAR3 cells, the mesenchymal characteristics were reversed, which is confirmed by the upregulated E-cadherin level and downregulated Snail and vimentin. These results suggested that FOXA1 was a prognostic biomarker in EOC and could promote the EMT progress of EOC cells.

It was demonstrated that FOXA1 have a directly and/or indirectly role in the regulation of EMT occurrence. Our findings were consistent with the results in other research [41, 42]. For example, Badve et al. implied that the concentration of FOXA1 varied at different stages in the course of tumor progression with a significant correlation with drug resistance and poor prognosis in both breast and prostate tumors [30]. In addition, FOXA1 was upregulated in malignant ovarian cancer tissues with substantial differences between the early and advanced stages [26]. On the other hand, researchers also pointed out that FOXA1 might suppress tumorigenesis in some cancer types by inhibiting EMT. For example, Song et al. suggested that the inhibition of FOXA1 expression could provoke the activation of EMT in pancreatic cancer [43]. Zhang et al. found that a high level of FOXA1 inhibited cell invasion and proliferation in breast cancer [44]. In another breast cancer research, FOXA1 was found to downregulate EMT-associated markers, including E-cadherin, ZEB2, and vimentin, eventually preventing EMT progression [45]. Likewise, similar outcomes were also observed in liver cancer, nasopharyngeal cancer, gastric cancer, prostate cancer, and colorectal cancer, in which the EMT process seemed to be reversed due to the presence of FOXA1 [46]. The possible explanation could be that FOXA1, as a strong activator of E-cadherin transcription, could serve as a tumor suppressor gene which could possibly reverse EMT by increasing E-cadherin expression, restoring the epithelial phenotype of the cancer cells [38]. Taken together, FOXA1 might influence the viability, proliferation, and invasion of tumor cells by affecting different signaling pathways in different cancers, leading to various effects on EMT.

Activated CTGF/TGF-*β* pathway leads to a loss of adhesion between cells, accelerating the development of EMT and metastasis in tumors [47]. There are many researches on CTGF pointing out its direct or indirect role in facilitating tissue fibrosis or profibrotic TGF-*β*1 activity [35, 40, 42, 44]. Persistent activation of TGF-*β* pathway is associated with malignancies of cancers. Burns et al. have comprehensively explained the mechanisms of CTGF in potentiating and enhancing TGF-*β* signaling either by increasing the affinity between TGF-*β* molecule and its receptor through physical interactions in the extracellular matrix or by abrogating the negative TGF-*β* feedback loop (Smad7) following binding to TrkA [20]. The present study proves the interaction between FOXA1 and the promoter of CTGF, suggesting that FOXA1 can increase the transcription of CTGF. Transcribed CTGF enters the ECM functions as a strong enhancing mediator of the TGF-*β* signaling pathway. This helps to explain the reason why TGF-*β* signaling was significantly enhanced in OVCAR3 cells, especially in those stimulated with the TGF-*β* cytokine. Furthermore, it has been shown that the persistence and severity of fibrosis caused by simultaneous injection of both TGF-*β* and CTGF far more exceed that caused by the injection of individual TGF-*β* or CTGF on its own. In the study of renal interstitial fibrosis, CTGF is an important cytokine that affect the prognosis and progression of disease [48]. Congruently, our study found that FOXA1, CTGF, and TGF-*β* are intercorrelated. Overexpression of FOXA1 is highly correlated with the elevation of endogenous CTGF and cleaved-TGF-*β* in OVCAR3. This is because in cancer cells, amplified FOXAl upregulates the expression of CTGF, which elicits prolonged activation of the CTGF/TGF-*β* pathway. Thereby, many important TGF-*β*-pathway-induced elements associated with EMT features, cell proliferation and invasion, and ECM remodeling are expected to be modulated correspondingly.

LiCl, as an inhibitor for GSK-3, has been reported to inhibit EMT efficiently [48]. In accordance with the previous findings, our study showed that LiCl leads to downregulations of FOXA1, CTGF, and cleaved-TGF-*β* in OVCAR3 cells, indicating the potential role of LiCl in controlling EMT progression in EOC. Further analysis demonstrated that LiCl combined with siRNA-FOXA1 silencing exerted a more robust effect on inhibiting EMT in OVCAR3. In addition, our study has some limitations. For example, to assess the validity of the functional experiments, two cell models should be used. The upstream regulatory mechanism of FOXA1 was not clear. In our future studies, we will delve into these directions.

Tumor microenvironment, especially tumor immune microenvironment, plays a vital role in accelerating tumor progression. In our study, we predicted that FOXA1 is involved in immune regulation-related pathways using GSEA. Moreover, FOXA1 was positively correlated with MHC genes, immunosuppressive genes, immune activating genes, and chemokine receptors, indicating a pivotal role of FOXA1 in tumor immunomodulatory function, especially in TCGA-OV cohort. Since the immunosuppressive microenvironment is not conducive to the efficacy of immune checkpoint inhibitors, we speculated that patients with high expression of FOXA1 are resistant to immunotherapy. Through our analysis of immunotherapy datasets, we found that FOXA1 expression was higher in PD/SD group than that in CR/PC group in GSE135222 cohort and Checkmate cohort. High expression of FOXA1 predicted poorer survival status of patients undergoing immunotherapy. These results indicated that patients with high FOXA1 expression may be resistant to immunotherapy. Further analysis also suggested that patients with high expression of FOXA1 may be resistant to most anticancer drug treatments, such as camptothecin, vinblastine, and cisplatin.

## 5. Conclusions

In our study, we conducted a comprehensive assessment of FOXA1, revealing a potential role of FOXA1 as an indicator of patient prognosis and molecular mechanism of FOXA1 to promote EMT by regulating CTGF/TGF-*β* pathway in ovarian cancer. We also predicted that FOXA1 was involved in the formation of tumor immunosuppressive microenvironment. In addition, EOC patients with high FOXA1 expression may be resistant to immunotherapy and most anticancer drug treatments. Targeting FOXA1 may become a potential treatment of EOC patients.

## Data Availability

The datasets generated and/or analyzed during the current study are available from the corresponding author upon reasonable request.

## Abbreviations

EMT: Epithelial-mesenchymal transition
EOC: Epithelial ovarian cancer
LiCl: Lithium chloride
MMPs: Matrix metallopeptidases
bHLH: Basic helix-loop-helix
FOXA1: Forkhead-box A1
OS: Overall survival
FBS: Fetal bovine serum
DMEM: Dulbecco's modified Eagle's medium
ANOVA: Analysis of variance
TCGA: The Cancer Genome Atlas
ACC: Adrenocortical carcinoma
BLCA: Bladder urothelial carcinoma
BRCA: Breast invasive carcinoma
COAD: Colon adenocarcinoma
DLBC: Diffuse large B-cell lymphoma
ESCA: Esophageal carcinoma
KICH: Kidney chromophobe
KIRC: Kidney renal clear cell carcinoma
LGG: Low-grade glioma
LIHC: Liver hepatocellular carcinoma
LUAD: Lung adenocarcinoma
LUSC: Lung squamous cell carcinoma
PFI: Progression-free interval
PRAD: Prostate adenocarcinoma
READ: Rectum adenocarcinoma
SKCM: Skin cutaneous melanoma
STAD: Stomach adenocarcinoma
TGCT: Testicular germ cell tumors
THCA: Thyroid carcinoma
UCEC: Uterine corpus endometrial carcinoma
UCS: Uterine carcinosarcoma.
